# Pathway-specific differences between tumor cell lines and normal and tumor tissue cells

**DOI:** 10.1186/1476-4598-5-55

**Published:** 2006-11-02

**Authors:** Adam Ertel, Arun Verghese, Stephen W Byers, Michael Ochs, Aydin Tozeren

**Affiliations:** 1 Center for Integrated Bioinformatics, School of Biomedical Engineering, Science and Health Systems, Bossone 714, Drexel University, 3143 Chestnut Street, Philadelphia, PA 19104, USA; 2 Lombardi Comprehensive Cancer Center at Georgetown University, 3800 Reservoir Road, Washington DC 20057, USA; 3 Division of Bioinformatics, Fox Chase Cancer Center, 333 Cottman Avenue, Philadelphia, PA 19111-2497, USA

## Abstract

**Background:**

Cell lines are used in experimental investigation of cancer but their capacity to represent tumor cells has yet to be quantified. The aim of the study was to identify significant alterations in pathway usage in cell lines in comparison with normal and tumor tissue.

**Methods:**

This study utilized a pathway-specific enrichment analysis of publicly accessible microarray data and quantified the gene expression differences between cell lines, tumor, and normal tissue cells for six different tissue types. KEGG pathways that are significantly different between cell lines and tumors, cell lines and normal tissues and tumor and normal tissue were identified through enrichment tests on gene lists obtained using Significance Analysis of Microarrays (SAM).

**Results:**

Cellular pathways that were significantly upregulated in cell lines compared to tumor cells and normal cells of the same tissue type included ATP synthesis, cell communication, cell cycle, oxidative phosphorylation, purine, pyrimidine and pyruvate metabolism, and proteasome. Results on metabolic pathways suggested an increase in the velocity nucleotide metabolism and RNA production. Pathways that were downregulated in cell lines compared to tumor and normal tissue included cell communication, cell adhesion molecules (CAMs), and ECM-receptor interaction. Only a fraction of the significantly altered genes in tumor-to-normal comparison had similar expressions in cancer cell lines and tumor cells. These genes were tissue-specific and were distributed sparsely among multiple pathways.

**Conclusion:**

Significantly altered genes in tumors compared to normal tissue were largely tissue specific. Among these genes downregulation was a major trend. In contrast, cell lines contained large sets of significantly upregulated genes that were common to multiple tissue types. Pathway upregulation in cell lines was most pronounced over metabolic pathways including cell nucleotide metabolism and oxidative phosphorylation. Signaling pathways involved in adhesion and communication of cultured cancer cells were downregulated. The three way pathways comparison presented in this study brings light into the differences in the use of cellular pathways by tumor cells and cancer cell lines.

## Background

Cell lines derived from tumors and tissues comprise the most frequently used living systems in research on cell biology. Limitations on the abundance of tissue samples necessitate the use of animal models and cell lines in the studies of tumor-related phenomena. Cancer cell lines have been extensively used in screening studies involving drug sensitivity and effectiveness of anti cancer drugs [1]. Other studies using cultured cells aimed at the determination of the phenotypic properties of cancer cells such as proliferation rates, migration capacity and ability to induce angiogenesis [2]. In other studies, human cultured cells were used to create tumors in the mice models [3].

Whether measurements on cell lines provide information about the metastatic behavior of cancer cells in vivo is currently under investigation. Unsupervised classification of gene expression profiles of cancer tissue and cancer cell lines result in separate clustering of cancer cell lines from tissue cells for both solid tumors and blood cancers [4]. Sets of genes responsible for differences between solid tumors and cell lines in their response to anti cancer drugs have been identified in the Serial Analysis of Gene Expression (SAGE) Database [5]. Most optimal cell lines to represent given tumor tissue types were determined with the use of a quantitative tissue similarity index [6]. Results were striking: only 34 of the 60 cell lines used in the analysis were most similar to the tumor types from which they were derived. The study provided valuable information about selection of most appropriate cell lines in pharmaceutical screening programs and other cancer research. In a more recent work Sandberg et al. [7] identified those gene function groups for which cell lines differed most significantly from tumors based on meta-analysis using Gene Ontology (GO). Genes involved in cell-cycle progression, protein processing and protein turnover as well as genes involved in metabolic pathways were found to be upregulated (an increase in expression reflected by mRNA transcript levels) in cell lines, whereas genes for cell adhesion molecules and membrane signaling proteins in cell lines were downregulated (a decrease in expression reflected by mRNA transcript levels) in comparison with tumors [7]. To build on this approach, functional enrichment analysis based on Kyoto Encyclopedia of Genes and Genomes (KEGG) pathways [8,9] can be used to illustrate causal relationships between genes (gene products). While GO is organized into hierarchical annotations in the context of normal cellular function, the KEGG database organizes the genes (gene products) into pathway reaction maps and functional complexes, including some disease-specific pathways.

The present study focuses on pathway specific differences in gene expression patterns between cancer cell lines and tumors as well as cancer cell lines and normal tissue and tumors and normal tissue. Extension of microarray data analysis to three-way comparison allows for the identification of gene expression patterns unique to cell lines. Such patterns might have arisen due to factors related to the cell culture environment. We used publicly accessible microarray data available for normal and cancer tissues and associated NCI60 cell lines in a pathway-specific quantitative analysis of gene expression profiles. A dominant theme that emerged from our analysis was that pathway-specific gene expression differences between cancer cell lines and cancer tissue were similar both in magnitude and direction to corresponding differences between cell lines and normal tissue cells. Cell cycle associated differences between normal and tumor tissue were amplified in cell lines. Results on metabolic pathways suggested an increase in the velocity of RNA and DNA production and increased flow of metabolites in the oxidative phosphorylation pathway. On the other hand, a small fraction of significantly altered genes in tumor-to-normal comparison had similar expressions in cancer cell lines and tumor cells. These genes were tissue-specific and were positioned sparsely along multiple pathways.

## Materials and methods

### Microarray datasets

Microarray datasets used in this study consisted of the publicly accessible gene expression profile dataset for NCI60 cell lines [10] and similar data for a panel of tumors and normal tissue samples [11]. This dataset contains measurements obtained using the Affymetrix Hu6800 arrays (Table 1). The tissue types considered in this study (breast, CNS, colon, prostate, ovary, and renal tissue) were restricted to those where the microarray results were available for normal and tumor tissue as well as corresponding cell lines. MDA-MB-435 and MDN cell line samples were excluded from these datasets because their tissue of origin, previously thought to be breast, is now suspect [6].

**Table 1:** Microarray data presented by Staunton et al. [10] and Ramaswamy et al. [11] used in the three way comparison of gene expression patterns in cell lines, tumors and normal tissue.

	Cell lines*	Normal tissue**	Tumor tissue**	**Array**
**Breast**	6	5	10	Affymetrix
**CNS**	6	5	20	Affymetrix
**Colon**	7	10	9	Affymetrix
**Ovary**	6	4	9	Affymetrix
**Prostate**	2	7	7	Affymetrix
**Renal**	8	11	8	Affymetrix

**Sum**	35	42	63	

* Data obtained from Staunton et al [10]

** Data obtained from Ramaswamy et al [11]

### Quality of probe set annotations

Quality of the Hu6800 GeneChip annotation was assessed because this platform is several versions away from current human microarrays. While the Hu6800 design is old and probe designs have since been greatly improved, the quality of probe annotation is maintained through regular updates by Affymetrix. The annotations used in this study are based on a July 12^th ^2006 update of Affymetrix annotations according to the March 2006 (NCBI Build 36.1) version of the human genome. A comparison was done between gene annotations for the Hu6800 GeneChip obtained from Webgestalt (web-based gene set analysis toolkit) [12] and from the Affymetrix website on August 7^th^, 2006. Out of the 7129 probesets on the chip, 6058 had the same annotations from both Webgestalt and Affymetrix. Of the remaining 1071 probesets, 692 were not annotated, 288 were annotated in the Affymetrix list but not in Webgestalt, 28 were annotated in Webgestalt but not Affymetrix, and 63 (~1%) probesets had conflicting annotations in Webgestalt and Affymetrix. Only 42 (~0.70% of all genes) genes belonging to any known KEGG pathway had discrepancies between Webgestalt and Affymetrix. While there were very few probes with discrepant annotations in any given pathway, this list of 42 probes was enriched for Antigen processing and presentation, Natural killer cell mediated cytotoxicity, Cell adhesion molecules (CAMs), Type I diabetes mellitus, and SNARE interactions in vesicular transport pathways. A review of this probe list revealed that discrepancies were merely due to updates and minor revisions to the official gene symbol that may reflect increased understanding of these genes functions. Genes associated with KEGG pathways represent a subset of well-studied and sequenced genes. Overall, the probe sets of genes belonging to KEGG pathways have well established and reliable annotations on the Hu6800 GeneChip. Annotations retrieved from Webgestalt were used for the remainder of the analysis.

### Normalization

Gene expression data was normalized for each tissue type by computing the Robust Multichip Average (RMA) [13,14] directly from the Affymetrix .CEL files for cell line, tumor, and normal samples. RMA consists of three steps: a background adjustment, quantile normalization and finally summarization. Quantile normalization method utilizes data from all arrays in an experiment in order to form the normalization relation [13,14] RMA generated expression measure is on the log base 2 scale.

Normalized data was generated using the Bioconductor (package for R) [15] implementation of RMA. R 2.3.1 [16] was first installed on an Intel Xeon machine running a Windows Professional Operating System. The Biobase 1.10.1 (dated 20 June 2006) package which contains the base functions for Bioconductor was installed by accessing the getBioC.R script directly from the Bioconductor website [17]. The "readaffy" command was used to load all .CEL files for a single tissue type. The RMA expression measures for each tissue type were computed using the "rma" function with default settings, including the Perfect Match Adjustment Method setting as Perfect Match Only so that expression signal calculation was based upon the perfect match values from each probe set as described in [13]. The RMA computed expression values were written out to a comma separated text file.

The resulting expression values for each sample were checked against the average expression across cell line, tumor, and normal populations by calculating their correlation coefficients. Two anomalous samples (one normal tissue sample from colon and one tumor sample from prostate) were identified having correlations well outside the remaining population (R < 0.9) and removed; RMA for those tissues was recomputed excluding the suspect samples. The RMA generated gene expression data for the Affymetrix chips was clustered using a hierarchical clustering algorithm with Pearson correlation coefficient as the distance metric using average linkage using TIGR MeV Version 3.1. For each of the six tissues under consideration, the cell line samples clustered together in a single branch distinct from the branches containing tumor and normal tissue samples. This result confirmed that all the cell line samples have characteristics that are significantly different from the tumor tissue.

### Significance analysis for gene expression

The Significance Analysis of Microarray Data (SAM) implementation [18] in the TIGR MeV Version 3.1 software [19] was used to identify those genes that had statistically significant differences in expression between tumor samples, cell lines, and normal tissue. SAM analysis was performed using all default parameters and adjusting the delta-value to obtain a maximum number of genes while maintaining a conservative false discovery rate of zero. A list of significant genes was identified for cell line-tumor cell line-normal and normal-tumor combinations for each of the six tissue types. When the set of significant genes was deleted from the microarray data, clustering analysis based on the remaining genes interspersed microarray datasets for cell lines with corresponding datasets for tissue.

### Identification of significantly altered pathways

Two different methods were used for identifying significantly altered pathways. First, Kyoto Encyclopedia of Genes and Genomes (KEGG) pathways [8,9] were identified as significantly altered by performing a functional enrichment analysis on genes identified as significant by SAM analysis. The analysis was carried out using the Webgestalt system [12], comparing significant genes obtained by SAM against all genes in the Affymetrix HU6800 array, for each comparison under study. A p-value for pathway enrichment was obtained using the hypergeometiric test documented in [12]. Four different p-value cutoffs (0.001, 0.01, 0.05 and 0.1) were used in order to assess the dependence of the significant pathway identification on p value. This process was also applied to subsets of significant genes, for example, the intersection of significant genes from (CL - N) and (T - N).

A second method was applied to KEGG pathway genes in order to detect changes that were not apparent on a single-gene basis. For this method, KEGG pathways were deemed significantly altered if at least 80% of the genes for that pathway contained on the HU6800 array were shifted in the same direction for a given comparison. For each of the six tissues, three-way comparisons were performed between averaged cell line, tumor, and normal samples. Similar examples of how significant changes in functional pathways are revealed by a population of related genes that are not evident from observations of a single gene are found in [20,21].

## Results

### Significant genes

This article presents a pathway-specific analysis of gene expression profile differences between cancer cell lines and normal and tumor tissue. The microarray data used in the three-way comparison of gene expression profiles covered breast, CNS, colon, ovary, prostate, and renal tissue (Table 1). Gene expression profiles of cancer cell lines derived from this data clustered together in a branch exclusive of tumor and normal tissue (3) within each tissue type and for all tissue types combined. Lists of significant genes (SAM genes) were determined using SAM analysis from the microarray data pairs of cell lines and tumors (CL - T), cell lines and normal tissue (CL - N) and tumor and normal tissue (T - N) for each of the six tissue types under consideration. Table 2 provides a summary of the numbers of significant genes for the three-way comparison. The table shows that the significant genes for (CL - T) and (CL - N) pairs ranged in numbers from low hundreds to thousands, depending on the tissue type. Significant genes for (T - N) pairs were lower in number than those for (CL - T) and (CL - N) pairs in all six tissues under consideration. Downregulation of significant genes was a trend in (T - N) comparisons while a majority of SAM genes were upregulated in cell lines compared to tumor and normal (CL - T; CL - N). Moreover, an overwhelming majority of the SAM genes in (T - N) comparison were not found as significantly altered in (CL - T) comparisons. The gene set (T - N) - (T - N ∩ CL - T) listed in Table 2 shows a vast majority of SAM genes in (T - N) comparison are not significantly altered in expression in (CL - T) comparison, suggesting that cancer cell lines may be good representation models for tumor cells in gene expression profile studies. On the other hand, the set (CL - T) contains many more genes than the (T - N) comparison, revealing that cancer cell lines have a large number of genes that are significantly altered in expression compared to tumor cells. The same trend holds true when cell lines are compared with normal tissue cells. These results indicate that global gene expression profiles of cultured cancer cell lines contain significantly different gene expression patterns compared to the corresponding profiles for normal and tumor tissue.

**Table 2:** Number of significant genes identified by SAM in comparisons of cell line-to-tumor (CL - T), cell line-to-normal (CL - N), and tumor-to-normal (T - N) comparisons.

Comparison	Breast	CNS	Colon	Ovary	Prostate	Renal	Common Genes
CL-T (upregulated %)	572 (66%)	576 (86%)	503 (62%)	603 (41%)	190 (94%)	1637 (44%)	51
CL-N (upregulated %)	269 (61%)	560 (72%)	983 (63%)	225 (62%)	469 (72%)	2047 (45%)	29
T-N (upregulated %)	243 (10%)	153 (61%)	166 (45%)	94 (14%)	30 (0%)	65 (0%)	0
CL-T ∩ CL-N	132	328	431	145	164	1481	16
(T-N) - (T-N ∩ CL-T)	236	138	143	83	26	43	0
(T-N ∩ CL-N) - (T-N ∩ CL-N ∩ CL-T)	31	43	64	26	9	28	0

The percentage of upregulated genes is shown in parentheses for cell line-to-tumor (CL - T), cell line-to-normal (CL - N), and tumor-to-normal (T - N) comparisons. The intersection (CL - T ∩ CL - N) contains genes that were altered in cell lines compared to both normal and tumor tissue, representing expression profiles that are specific to cell lines. The set (T - N) - (T - N ∩ CL - T) contains SAM genes in the (T - N) comparisons that are not significantly altered in (CL - T) comparisons. The set (T - N ∩ CL - N) - (T - N ∩ CL - N ∩ CL - T) contains genes significantly altered in both cell lines and tumors relative to normal tissue (T - N; CL - N) but with no significant difference between cell lines and tumor tissue (CL - T); tumor-specific expression profiles that may be adequately modeled by cell lines.

SAM genes common in (CL - T) comparisons for all six tissues were all upregulated. Table 3 shows the list of 51 significant genes in (CL - T) comparisons that are common to the six tissue types under consideration. In this list of 51 genes, the overrepresented KEGG pathways with a p-value cutoff of 0.01 are cell cycle, oxidative phosphorylation, proteasome, pyrimidine metabolism, and ubiquitin mediated proteolysis. The 18 genes shown in italics also appeared among 29 significant genes that were common to all (CL - N) comparisons. The 18 genes common to both lists again showed overrepresentation of cell cycle and ubiquitin mediated proteolysis pathways under a p-value cutoff of 0.01. Moreover these eighteen genes showed the same trend of upregulation in cell line-to-tumor (CL - T) and cell line-to-normal (CL - N) comparisons. No significant genes identified in the (T - N) comparisons were common to all six tissues.

**Table 3:** SAM genes that were upregulated in cell lines compared to tumors in all the 6 tissues considered in the study (CL - T).

**Gene Symbol**	**Gene Name**	**Kegg Pathway(s)**
ATP5B	ATP synthase, H+ transporting, mitochondrial F1 complex, beta polypeptide	Oxidative phosphorylation, ATP synthesis
ATP5G3	ATP synthase, H+ transporting, mitochondrial F0 complex, subunit C3 (subunit 9)	ATP synthesis, Oxidative phosphorylation
C1QBP	complement component 1, q subcomponent binding protein	(Immune Response)
CBX3	chromobox homolog 3 (HP1 gamma homolog, Drosophila)	N/A
CCNB1	cyclin B1	Cell cycle
CCT5	chaperonin containing TCP1, subunit 5 (epsilon)	N/A
*CDC20*	CDC20 cell division cycle 20 homolog (S. cerevisiae)	Ubiquitin mediated proteolysis, Cell cycle
*CDKN3*	cyclin-dependent kinase inhibitor 3 (CDK2-associated dual specificity phosphatase)	N/A
CHAF1A	chromatin assembly factor 1, subunit A (p150)	N/A
CKAP1	cytoskeleton associated protein 1	N/A
CKS1B	CDC28 protein kinase regulatory subunit 1B	N/A
*CKS2*	CDC28 protein kinase regulatory subunit 2	N/A
COX8A	cytochrome c oxidase subunit 8A (ubiquitous)	Oxidative phosphorylation
*CYC1*	cytochrome c-1	Oxidative phosphorylation
DNMT1	DNA (cytosine-5-)-methyltransferase 1	Methionine metabolism
DYNLL1	dynein, light chain, LC8-type 1	N/A
EBNA1BP2	EBNA1 binding protein 2	N/A
HMGB2	high-mobility group box 2	N/A
KIAA0101	KIAA0101	N/A
*KIF2C*	kinesin family member 2C	N/A
LMNB2	lamin B2	Cell communication
*MCM3*	MCM3 minichromosome maintenance deficient 3 (S. cerevisiae)	Cell cycle
*MCM4*	MCM4 minichromosome maintenance deficient 4 (S. cerevisiae)	Cell cycle
MCM7	MCM7 minichromosome maintenance deficient 7 (S. cerevisiae)	Cell cycle
*MRPL12*	mitochondrial ribosomal protein L12	N/A
*NDUFS8*	NADH dehydrogenase (ubiquinone) Fe-S protein 8, 23kDa (NADH-coenzyme Q reductase)	Oxidative phosphorylation
PAICS	phosphoribosylaminoimidazole carboxylase, phosphoribosylaminoimidazole succinocarboxamide synthetase	Purine metabolism
*PCNA*	proliferating cell nuclear antigen	Cell cycle
POLR2G	polymerase (RNA) II (DNA directed) polypeptide G	Purine metabolism, RNA polymerase, Pyrimidine metabolism
*PRMT1*	protein arginine methyltransferase 1	Selenoamino acid metabolism, Nitrobenzene degradation, Aminophosphonate metabolism, Tryptophan metabolism, Histidine metabolism, Androgen and estrogen metabolism, Tyrosine metabolism
PSMA1	proteasome (prosome, macropain) subunit, alpha type, 1	Proteasome
PSMB2	proteasome (prosome, macropain) subunit, beta type, 2	Proteasome
PSMB5	proteasome (prosome, macropain) subunit, beta type, 5	Proteasome
*PSMB6*	proteasome (prosome, macropain) subunit, beta type, 6	Proteasome
PSMD14	proteasome (prosome, macropain) 26S subunit, non-ATPase, 14	Proteasome
*RANBP1*	RAN binding protein 1	N/A
SFRS9	splicing factor, arginine/serine-rich 9	N/A
SNRPA	small nuclear ribonucleoprotein polypeptide A	N/A
SNRPB	small nuclear ribonucleoprotein polypeptides B and B1	N/A
SNRPC	small nuclear ribonucleoprotein polypeptide C	N/A
*SNRPD2*	small nuclear ribonucleoprotein D2 polypeptide 16.5kDa	N/A
SNRPD3	small nuclear ribonucleoprotein D3 polypeptide 18kDa	N/A
SNRPE	small nuclear ribonucleoprotein polypeptide E	N/A
SNRPF	small nuclear ribonucleoprotein polypeptide F	N/A
SNRPG	small nuclear ribonucleoprotein polypeptide G	N/A
*TCEB1*	transcription elongation factor B (SIII), polypeptide 1 (15kDa, elongin C)	Ubiquitin mediated proteolysis
*TUBG1*	tubulin, gamma 1	N/A
TXNRD1	thioredoxin reductase 1	Pyrimidine metabolism
TYMS	thymidylate synthetase	Pyrimidine metabolism, One carbon pool by folate
*UBE2C*	ubiquitin-conjugating enzyme E2C	Ubiquitin mediated proteolysis
*UBE2S*	ubiquitin-conjugating enzyme E2S	N/A

SAM genes shown in italic also belonged to cell lines and normal tissue comparison. There were no downregulated genes common to all tissue types in cell line-tumor (CL - T) comparisons.

### Significant pathways

**Figure 1:**
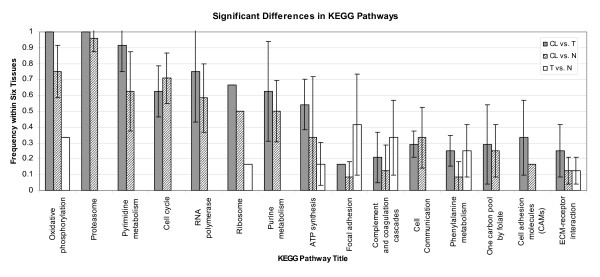
**KEGG pathways identified to be significantly altered in cell lines and tumors (CL - T), cell lines and normal tissue (CL - N), and tumor and normal tissue (T - N) comparisons**. The term frequency shown in the figure is defined as the ratio of tissue types for which a pathway identified as significantly altered to the total number of tissue types (6). KEGG pathways were identified as significantly altered by using a hypergeometric test with a p-value cutoff. The minimum number of SAM genes in each significantly altered pathway has been set to two. The error bars indicate the standard deviation of frequency for different p- value cutoffs (p = 0.001, 0.01, 0.05 and 0.1).

KEGG pathways whose gene expression profiles differed significantly in (CL - T), (CL - N), and (T - N) pair comparisons were identified using a hypergeometric test as described in the Methods section. Figure 1 shows the most frequently observed KEGG pathways with altered gene expression profiles for (CL - T), (CL - N) and (T - N) pairs for breast, CNS, colon, ovary, prostate, and renal tissue. Cell cycle and a number of metabolic and transcription-related pathways emerged as significantly altered in almost all (CL - T) and (CL - N) comparison pairs. Cellular pathways that were significantly altered in cell lines compared to tumor cells and normal cells of the same tissue type in at least two tissue types included cell cycle, oxidative phosphorylation, purine and pyrimidine metabolism, proteasome, ribosome, and RNA polymerase. The most striking difference between cell lines and tumor tissue in Figure 1 is in the oxidative phosphorylation pathway. Oxidative phoshorylation is the final stage of cellular metabolism following glycolysis and the citric acid cycles. The loss of cancer cell dependence on oxidative metabolism may be an important factor in the development of tumors [22]. ECM-receptor interaction, which is thought to affect cell migration, appeared with more subtle differences between all comparisons (CL - T), (CL - N), and (T - N). This may reflect more tissue-specific composition of the migration machinery utilized in tumor cell invasion.

**Figure 2:**
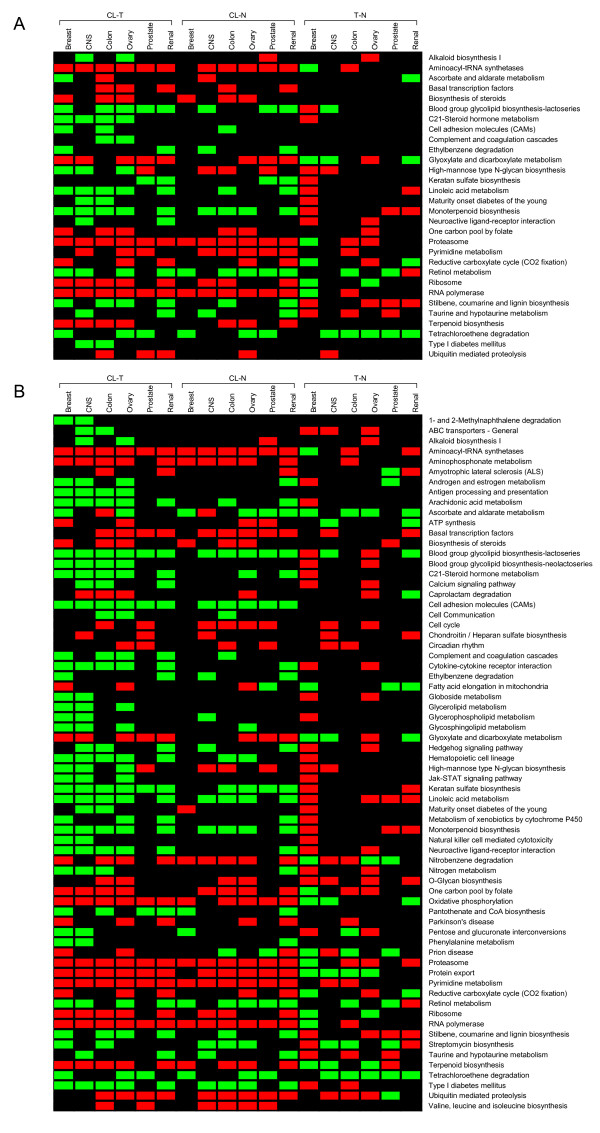
**A module map showing the direction of regulation of cellular pathways that were identified as significantly altered in cell lines compared to tumor tissue (CL - T) in at least 2 of the 6 tissues considered in this study**. In (a), a pathway is deemed significantly altered if at least 80% of the genes in the pathway are shifted in a common direction. In (b), a pathway is deemed significantly altered if at least 70% of the genes in the pathway are shifted in a common direction. The color red indicates an upregulated pathway, the color green indicates a downregulated pathway, and the color black indicates that the pathway was not significant in that comparison.

Next we used pathway-specific analysis to identify up- and downregulation patterns in three-way comparisons. Figure 2 provides module maps showing the direction of regulation of KEGG pathways that were identified to be significantly different in at least 2 tissue types in (CL - T) comparisons. The pathways presented in Figure 2a were deemed significantly altered if the average gene expression between two conditions was altered in the same direction for at least 80% of the genes in the pathway. This criterion captured seven of the significant pathways from Figure 1 along with 23 additional pathways. The Figure 2a indicates a high degree of correlation in the direction of Aminoacyl-tRNA synthetases, Monoterpenoid biosynthesis, Proteosome, and RNA polymerase pathway shifts in cell line – tumor and cell line – normal comparisons. Many more pathways appear to be significantly altered in the module map if the criterion for percentage of genes altered in the same direction is reduced from 80% to 70% (Figure 2b). These two module maps illustrate how extensive the pathway alterations are in cell lines compared to tumor and normal tissue (CL - T; CL - N).

The pathway-specific results on cell line-tumor microarray data comparisons presented in this study are in agreement with the results recently published by Sandberg et al. [7] on the gene expressions patterns associated with gene ontology categories in cell lines and tumors. These authors have used the same microarray databases used in our study and reached highly similar conclusions on the directions of difference between cell lines and tumors along equivalent pathways and gene ontology categories. Table 4 provides a comparison of the KEGG pathways (from Figure 1) against the most related gene ontology categories from Sandberg et al. [7]. KEGG pathways for complement and coagulation cascade and phenylalanine metabolism passed the significance criteria based on the (T - N) comparison in our study but we could not located the corresponding GO categories in the Sandberg et al. study on cell lines vs. tumor tissue.

**Table 4:** Comparison of results obtained from this study with those based on Gene Ontology Processes by Sandberg et al. [7]

**KEGG Pathway**	**Related GO category**	**Direction of regulation in cell lines with respect to tumors**	
		**This study**	**Gene Ontology Study [7]**
ATP synthesis	ATP synthesis coupled proton transport *(GO:0015986)*	↑	↑
Cell cycle	Cell cycle *(GO:0007049)*	↑	↑
One carbon pool by folate	Nucleotide biosynthesis *(GO:0009165)*	↑	↑
Oxidative phosphorylation	Oxidative phosphorylation *(GO:0006119)*	↑	↑
Proteasome	Ubiquitin-dependent protein catabolism *(GO:0006511)*; Modification-dependent protein catabolism *(GO:0019941)*	↑	↑
Purine metabolism	Purine nucleotide metabolism *(GO:0006163)*	↑	↑
Pyrimidine metabolism	Nucleobase, nucleoside, nucleotide and nucleic acid metabolism *(GO:0006139)*	↑	↑
Ribosome	Protein biosynthesis *(GO:0006412)*	↑	↑
RNA polymerase	Nucleobase, nucleoside, nucleotide and nucleic acid metabolism *(GO:0006139)*	↑	↑
Cell adhesion molecules (CAMs)	Cell adhesion *(GO:0007155)*	↓	↓
Cell communication	Cell adhesion *(GO:0007155)*	↓	↓
Complement and coagulation cascade	Complement activation *(GO:0006956)*	↓	N/A
ECM-receptor interaction	Cell adhesion *(GO:0007155)*	↓	↓
Focal Adhesion	Cell adhesion *(GO:0007155)*	↓	↓
Phenylalanine metabolism	Phenol metabolism *(GO:0018958)*	↓	N/A

The symbol [↑] indicates upregulation in cell lines with respect to tumors and [↓] indicates downregulation in cell lines with respect to tumors (CL - T).

### Gene expression changes in metabolic pathways

**Figure 3:**
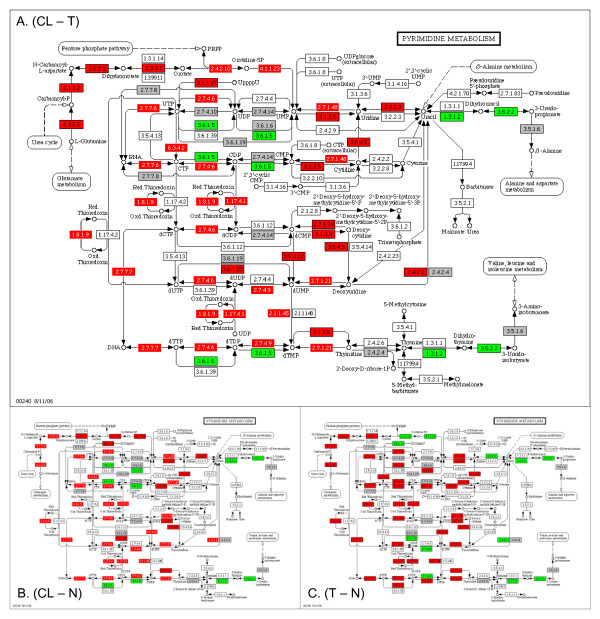
**KEGG pyrimidine metabolism diagram**. Gene expression shifts are projected from comparisons of cell line-to-tumor (CL - T), cell line-to-normal (CL - N), and tumor-to-normal (T - N) comparisons averaged over all six tissues. The color red indicates upregulated genes, green indicates downregulated genes and grey indicates the genes that are not on the microarray. Uncolored genes are not in the organism-specific pathway for Homo sapiens. A gene is identified as upregulated (downregulated) if its gene expression value averaged over 6 tissue types were greater (or lesser) in cell lines compared to tumor or normal tissue. Colored genes with white lettering were also identified with SAM in at least two tissues.

Metabolic pathways such as oxidative phosphorylation, pyrimidine and purine metabolism account for some of the most significant alterations among the three-way comparisons. The alterations in the oxidative phosphorylation pathway were discussed briefly in the previous section. Purine and pyrimidine metabolic pathways synthesize the nucleotides that make RNA and DNA. All of the nitrogens in the purine and pyrimidine bases (as well as some of the carbons) are derived from amino acids glutamine, aspartic acid, and glycine, whereas the ribose and deoxyribose sugars are derived from glucose. Figure 3 shows the KEGG diagram of pyrimidine metabolism with the expression values (averaged over six tissues) overlaid for (CL - T) (3a), (CL - N) (3b), and (T - N) (3c) comparisons. This KEGG pathway is altered with upregulated expression for a majority of genes in cell lines and tumors when compared to normal tissue. The increased levels of pyrimidine metabolism gene expression are most pronounced in cell lines (Fig 3a). A predicted increase in the velocity of RNA and DNA base production in cell lines is consistent with trends of increasing rates of cell division observed in cell cultures [23]. The observation that nucleotide metabolism accelerates in cancer has been discussed in the literature. Development of pyrimidine and purine analogs as potential antineoplastic agents evolved from an early presumption that cancer is a disease of uncontrolled growth and nucleic acids are involved in growth control [24].

### Gene expression pattern changes in cell cycle

**Figure 4:**
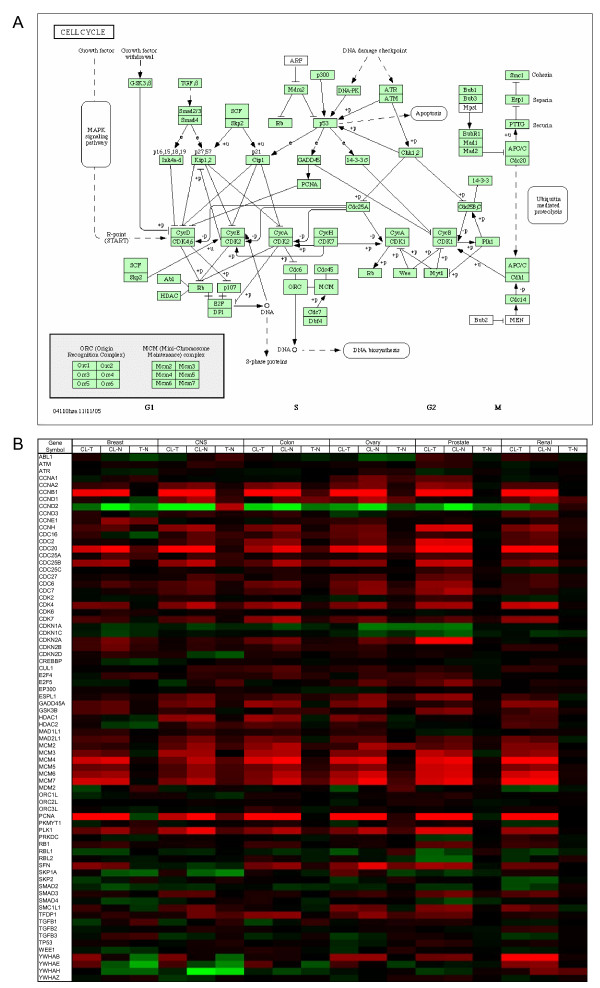
**KEGG cell cycle diagram**. Genes are shown (a) in a pathway map with genes specific to homo-sapiens shaded light green and (b) tabulated with a color map showing average gene expression shifts for samples within the six tissues. Red indicates a positive change and green indicates a negative change in average RMA value for the respective cell line-tumor (CL - T), cell line-normal (CL - N), and tumor-normal (T - N) comparisons, with color scale limits set to -2 and +2.

In contrast to the pyrimidine metabolism pathway discussed above, the gene expression alterations along the cell cycle pathway appear to be more complex and tissue-specific. Figure 4a shows the KEGG diagram of cell division cycle with genes specific to Homo sapiens shaded light green. Figure 4b shows the extent of alteration of these genes in the three-way comparisons for each tissue type with a graded color map representing maximum upregulation in red and maximum downregulation in green.

Perhaps the most obvious feature of this color map is how subtle the changes in (T - N) comparisons are relative to (CL - T) and (CL - N) comparisons in all six tissues under consideration. Genes such as CCNA2, CCNB1, CDC20, CDK4, and MDM2 through MDM7 are consistently upregulated in cell lines compared to tumors and normal tissue. On the other hand, genes such as CCND1, CCND3, CDC16, and CDK2 do not exhibit quickly a recognizable pattern. A multitude of gene expression profiles in cell cycle may point towards the same disease process.

### SAM genes common to cancer cell lines and tumor cells

It is of interest to cell biologists to identify similarities between cancer cell lines and tumors. Towards that goal, one can determine the list of SAM genes belonging to both (T - N) and (CL - N) comparisons but do not appear to be significant in (CL - T) comparison. This list is shown in Table 5 for all six tissues under consideration. Table 5 gives an indication of the size of the SAM gene subsets that are preserved and commonly regulated in cell lines and tumors but not in normal tissues. The list of genes in Table 5 comprises mostly downregulated genes for breast, colon, ovary, prostate, and renal tissue, with CNS as the only exception. When these lists were projected onto KEGG pathways, the probability of enrichment score could not be used as an indication that the pathways are similar because KEGG pathways that include genes from these lists also included SAM genes from (CL - T) comparisons. In conclusion, it was not possible to assert pathway similarity with statistical confidence using this analysis.

**Table 5:** Genes that were identified by SAM in both (T - N) and (CL - N) comparisons but not in (CL - T) comparisons; (T - N ∩ CL - N) – (T - N ∩ CL - N ∩ CL - T).

**Breast**		**CNS**		**Colon**		**Ovary**		**Prostate**		**Renal**	
**UP**	**DOWN**	**UP**	**DOWN**	**UP**	**DOWN**	**UP**	**DOWN**	**UP**	**DOWN**	**UP**	**DOWN**
GALNS	APP	ACTB	ATP5O	ARD1A	ADH1B	MCM2	ACTG2		APOD		ADH1B
GP9	AQP1	CPSF1	COX7A1	ARPC1B	BRD2		AEBP1		CCND2		ALDH4A1
LCAT	ARHGEF6	DDX11	CTNNB1	BCAT1	C7		C7		CXCL12		ANPEP
RND2	ATP6V1B2	ECE1	GYPE	CCND1	CA2		CEBPD		KCNMB1		ASS
	BRD2	EEF1A1	ITGB7	CPNE1	CALCOCO2		CNN1		MATN2		ATP6V1B1
	CTNNB1	EEF1G	KIAA0513	CUL7	CASC3		DPYSL2		PTGDS		C7
	CXCL12	FRAP1	MEF2C	ERCC1	CES2		DUSP1		PTN		CLCNKB
	DUSP1	GNAI2	MRPS21	GPS1	CHGA		EGR1		SERPING1		ENG
	EGR1	GNB1	MYOM2	MDK	CLEC3B		FOS		SPARCL1		EPHX2
	EGR3	GNB2	PCP4	PDXK	CNN1		GYPC				FABP1
	IGFBP4	GPIAP1	PVALB	PEX6	CRYAB		IGFBP5				GATA3
	JUND	GPS1	S100A1	PHLDA2	CTNNB1		JUNB				GATM
	KHSRP	H3F3B	SEPP1	S100A11	CUGBP2		LMOD1				GPX3
	KIT	HNRPF	SERPINI1	TEAD4	DMD		LUM				GSTA2
	KRT15	KHDRBS1			DPYSL2		MYH11				HMGCS2
	KRT5	MAZ			FABP4		MYLK				HPD
	MXI1	NONO			FCGBP		NDN				KCNJ1
	MYH11	ODC1			FGFR2		NR4A1				MT1G
	NFIB	PCBP2			FHL1		PPAP2B				MT1X
	NSMAF	RAB7			GDI1		SEPP1				PAH
	PCBP1	RBM10			GPD1L		SERPINF1				PALM
	SERPINA3	RBM5			HMGCS2		SPARCL1				PCK2
	SNTB2	RHOB			HSD11B2		TNXB				PRODH2
	SOX9	SMARCA4			HSPA1A		ZBTB16				PTHR1
	SPARCL1	SRM			IL11RA		ZFP36				SERPINA5
	VWF	TRIM28			IL6R						TACSTD1
	ZFP36	TUBB			ITGA7						UGT2B7
		UFM1			ITPKB						UMOD
		YBX1			LMOD1						
					LPL						
					MAOA						
					NFIB						
					NR3C2						
					PCK1						
					PLN						
					PPAP2B						
					PPP1R1A						
					PRKCB1						
					SEPP1						
					SLC26A3						
					SMTN						
					SPIB						
					SRPX						
					TACR2						
					TGFBR3						
					TPM1						
					TPM2						
					TSPAN7						
					TUBA3						
					ZBTB16						

## Conclusion

Our study shows that a large portion of genes implicated in the emergence and progression of cancer have similar gene expression values in tumors and cancer cell lines indicating the value of cultured cell lines in cancer research. However, the pair-wise comparisons of gene expression profiles of CL, T, and N across all tissues illustrate that there are pronounced changes in gene expression specific to cell lines (CL - T; CL - N) that may not represent a disease process. This study also identified the signaling and metabolic pathways in cell lines that have distinctly different gene expression patterns than those associated with normal and tumor tissue. Pathway-specific gene expression changes in (CL - T) and (CL - N) comparisons were more consistent than (T - N) comparisons in the set of six tissues under consideration. Just as the gene expression changes in tumor – normal tissue comparison were largely tissue-specific, the significantly altered pathways among tumor – normal comparisons were limited to a small number of tissues. Functional enrichment analysis allows us to explore significant changes in pathways despite having heterogeneous changes in gene expression across different tissues. Cellular pathways that were significantly upregulated in cell lines compared to tumor cells and normal cells of the same tissue type included ATP synthesis, cell cycle, oxidative phosphorylation, purine, pyrimidine and pyruvate metabolism, and proteasome. Results on metabolic pathways suggested an increase in the velocity nucleotide metabolism and RNA production.

The dominant trend in the gene expression profiles along significantly altered pathways in cell lines appeared to be upregulation of genes when compared either to tumor or normal tissue. Exceptions included genes in the cell adhesion molecules, cell communication, and ECM-receptor interaction, focal adhesion, and complement/coagulation cascade pathways. The apparent downregulation of the complement/coagulation cascade in cell lines may be due to the heterogeneous mixture of cells in tumor samples including immune cells as well as tissue-specific cells.

The composition of the cell culture medium may be the reason why gene expression patterns that differentiate cancer cell lines from tumor tissue are similar to those patterns that differentiate between cell lines and normal tissue. Typical cell culture medium is replete with metabolites, growth factors, and cytokines, among others, for which cells normally must compete in vivo [24]. Multicellular interfaces with which tumor cells interact in vivo are not replicated for cells grown in cell culture plates [26-29]. The differences in environmental selection pressures may help explain the differential gene expression patterns between the tumor tissue and the cell lines. Our finding about the upregulation of oxidative phosphorylation in cell lines is supported by previous metabolic studies [30,31]. The documentation of gene expression differences along signaling and metabolic pathways is important in compound screening during the drug discovery process. Compounds may affect significantly altered pathways between cell lines and tumor tissue differently. Recent studies are taking advantage of the technological advances in microfluidics and tissue engineering to develop three-dimensional cell culture systems that aim simulating in vivo culture conditions. Whether cell lines can be made to mimic tumor cell gene expression patterns by altering the culture medium conditions is a question yet to be fully explored.
